# Primary ectopic parasellar craniopharyngioma: a case report

**DOI:** 10.1186/s12883-021-02368-5

**Published:** 2021-09-03

**Authors:** Xintao Cai, Zhixiang Sun, Yu Li, Dongqi Shao, Xialin Zheng, Yu Wang, Zhiquan Jiang

**Affiliations:** Department of Neurosurgery, The First Affiliated Hospital of Bengbu Medical College, 287 Changhuai Road, 233000 Bengbu, People’s Republic of China

**Keywords:** Craniopharyngioma, Parasellar, Endoscopic sinus surgery

## Abstract

**Background:**

Craniopharyngioma (CP) is a slow-growing, benign tumor of the central nervous system located within the sellar and suprasellar regions. The tumor may extend from the suprasellar region to other areas. CPs are generally believed to originate from squamous remnants of an incompletely involuted craniopharyngeal duct that also develops from Rathke’s pouch. Primary parasellar craniopharyngioma is a relatively rare tumor, and nasal endoscopy, computed tomography, and enhanced magnetic resonance imaging can be applied to better evaluate the invasiveness and characteristics of these tumors.

**Case presentation:**

We report a case of right parasellar craniopharyngioma in a 49-year-old female patient with a 10-day history of dizziness and blurred vision. Preoperative imaging examination revealed right parasellar space-occupying lesions, and the patient underwent transnasal neuroendoscopic resection of the right parasellar space-occupying lesion. The postoperative pathological result confirmed craniopharyngioma.

**Conclusions:**

Primary ectopic parasellar craniopharyngioma is a relatively rare tumor, and preoperative imaging examination can assist in the evaluation of tumor characteristics. However, the final diagnosis continues to depend on the histopathological results.

## Background

Craniopharyngioma (CP) is relatively rare, accounting for only 3 % of all intracranial tumors [1]; this condition is a benign epithelial tumor that arises from the embryologic squamous epithelial remnants of the craniopharyngeal duct or Rathke’s pouch. The majority of these tumors have both intra- and suprasellar components [2, 3]. Primary presentations outside this region are rare [4]. Craniopharyngiomas in abnormal sites have been reported, including the fourth ventricle [5], subsellar region [6], lateral ventricle [7], and frontotemporal epidural space [8]. On the other hand, there are related studies indicating that ectopic recurrence of craniopharyngioma may occur in the surgical path [9], and there have been related reports of spinal metastasis of craniopharyngioma in childhood [10]. Here, we report a case of primary right parasellar ectopic craniopharyngioma treated by nasal endoscopy.

## Case presentation

**Fig. 1:**
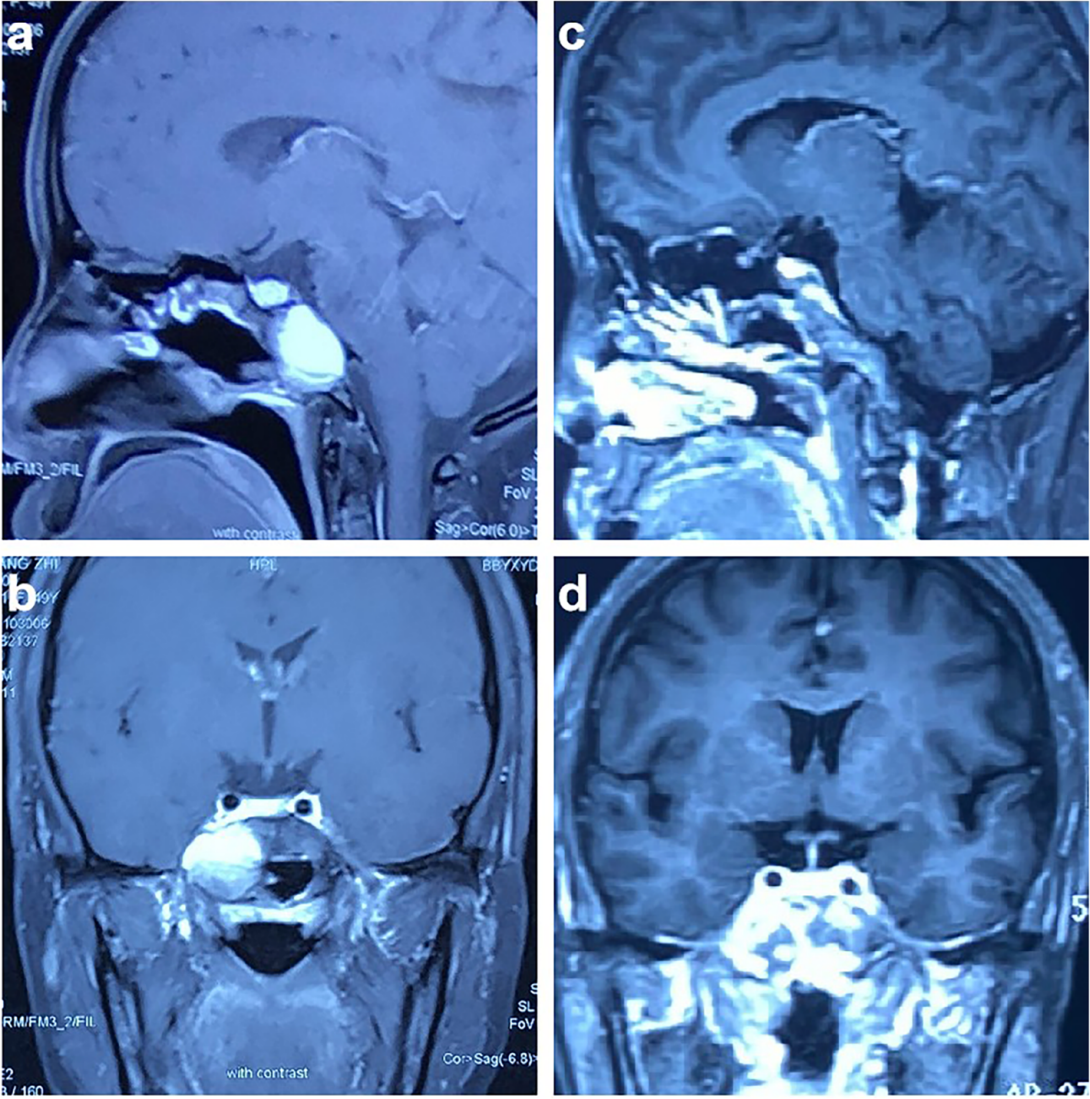
Enhanced magnetic resonance imaging (MRI) of the patient’s head. Preoperative enhanced MRI, including sagittal (**a**) and coronal (**b**) views, showed uneven T1 hyperintensity. A lesion with a length of approximately 33 mm was identified in the sphenoid sinus and right parasellar region, but no obvious enhancement was found. Six months after surgery, sagittal (**c**) and coronal (**d**) contrast-enhanced T1 MRI showed no residual tumor near the sella and no local recurrence

The patient was a 49-year-old female who complained of dizziness and blurred vision for more than 10 days. Neurological examination showed no significant abnormal signs, and the patient reported no history of irradiation, chemical exposure, or trauma. Brain magnetic resonance imaging (MRI) was performed in the outpatient department: Mixed-signal shadows were observed in the sphenoid sinus and right parasellar region, along with an uneven distribution of high T1 weighted image (WI) signal; T2 weighted image (WI) shows that the upper layer is of high intensity and the lower layer is of equal intensity, and there is a clear dividing line between them. The tumor showed no enhancement on contrast-enhanced MRI. The length of the lesion was approximately 33 mm (Fig. 1a and b).

**Fig. 2:**
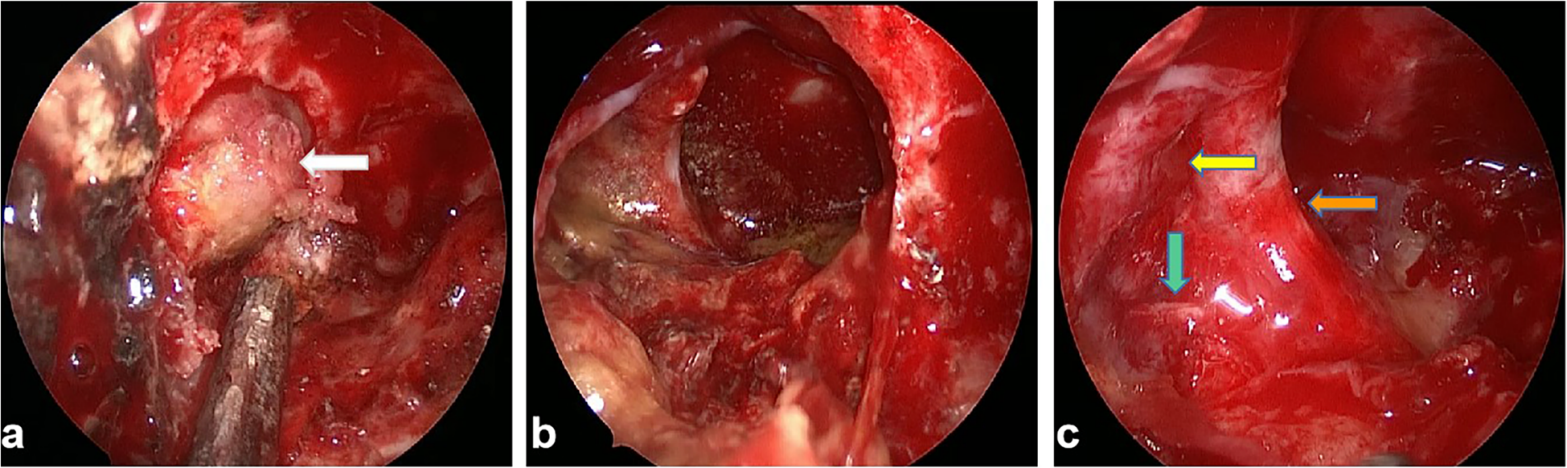
A nasal endoscopic view of the resection path for the space-occupying lesion of the right sella. (**a**) The size and shape of the tumor under direct visualization during endoscopy (white arrow). (**b**) The surgical field exposed after complete resection of the cyst wall and cyst contents. (**c**) In the anatomical region from which the exposed tumor was resected, the right internal carotid artery (orange arrow), the right trigeminal nerve (green arrow), and the cavernous sinus (yellow arrow) are visible

For further treatment, the patient was transferred from the Otolaryngology Department to the Neurosurgery Department of our hospital. Craniocervical computed tomography angiography(CTA) demonstrated a localized defect of the sphenoid and right occipital slope in the sphenoid sinus, with a low-density shadow in the corresponding area and local envelopment of the right internal carotid artery. Based on the preoperative imaging examination, the patient was diagnosed with a space-occupying lesion in the right parasellar region. The patient underwent transnasal neuroendoscopic resection of the right parasellar space-occupying lesion. We used a 0.01 % adrenaline saline tampon to push aside the turbinate and nasal septum on both sides to provide enough operating space for the endoscope. We then used a rigid 30° endoscope with an outside diameter of 4 mm (Karl Storz, Tuttlingen, Germany) to remove the tumor. Cystic tumor lesions were identified during the operation (Fig. 2a), along with local absence of bone in the right parasellar slope. The tumor was cut open, releasing an opaque, soy sauce–colored, opaque fluid and revealing brown deposits in the cyst. Complete resection of the cyst wall and contents was performed (Fig. 2b). The right internal carotid artery, the right trigeminal nerve, and the cavernous sinus were visible (Fig. 2c). Fluid gelatin (Johnson, New Jersey, USA) was injected into the bleeding site to stop the bleeding. The sellar floor was reconstructed with artificial dura mater (Tianxinfu, Beijing, China), and after this reconstruction, no cerebrospinal fluid leakage was observed. On the second day after the operation, no residual tumor was found on MRI. After 6 months, craniocerebral enhancement MRI was repeated, as shown in Fig. 1c and d; this scan showed no residual tumor near the sella and no local recurrence. After 12 months of follow-up, no tumor recurrence was observed.

**Fig. 3:**
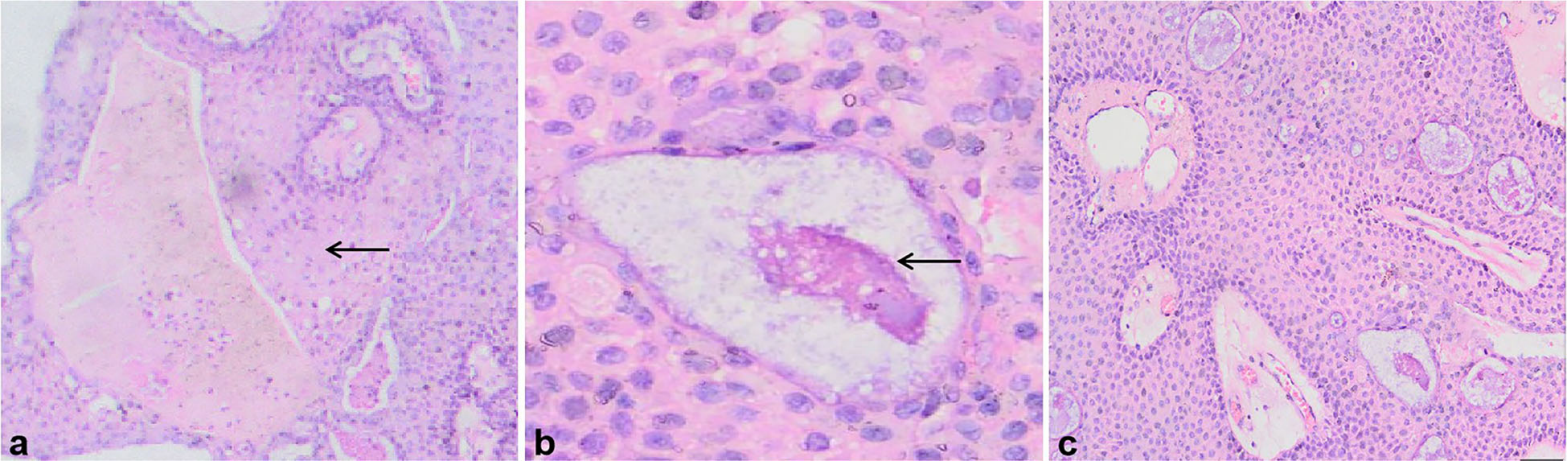
On histopathological examination, the tumor was diagnosed as craniopharyngioma. (**a**) Features of the tumor revealed partial keratinization (black arrow; hematoxylin and eosin staining; magnification: 40×; scale bar: 500 μm). (**b**) Cystic contents with significant cystic changes (black arrow; hematoxylin and eosin staining; magnification: 200×; scale bar: 500 μm). (**c**) The tumor was accompanied by a well-differentiated, irregularly arranged, squamous epithelial structure (hematoxylin and eosin staining; magnification: 200×; scale bar: 500 μm) surrounded by palisade columnar epithelium

The histopathological diagnosis of the tumor was CP, the tumor was characterized by partial keratosis (Fig. 3a), and the cystic contents of the tumor were accompanied by significant cystic changes (Fig. 3b).Moreover, with a well-differentiated, irregularly arranged squamous epithelium (Fig. 3c), surrounded by palisade-lined columnar epithelium.

## Discussion and conclusion

Craniopharyngioma is a benign tumor that occurs in the intrasellar or suprasellar region [11]. Ectopic craniopharyngioma can be divided into primary and secondary types, of which the latter is rare. Two different mechanisms of secondary craniopharyngioma have been described. The first mechanism is that the surgical removal of tumors leads to the contamination of healthy tissues by tumor cells. The second mechanism is the diffusion of craniopharyngioma cells through cerebrospinal fluid [12]. However, primary craniopharyngioma is rarer than secondary craniopharyngioma and arises in the absence of any previous surgery [11]. The infrasellar region (50 reported cases) is the most common abnormal site of craniopharyngioma, and the sphenoid sinus is the most common site of infrasellar craniopharyngioma [13]. The second most common extrasellar position is the cerebellopontine angle (13), followed by the frontotemporal region (4), fourth ventricle (3), pineal region (2) and corpus callosum (1) [14]. In this case, the craniopharyngioma was located in the right parasellar region; no craniopharyngioma in this area has been reported before.

CPs arise from the squamous epithelial remnants of Rathke’s pouch [15]. This pouch appears during the fourth gestational week. It is transformed into a canal that obliterates itself and disappears by resorption during the seventh gestational week. The resorption begins at the middle of the canal. This may explain why the residual Rathke’s pouch of the embryo often appears in nodules and other sites [16]. This also explains the phenomenon of craniopharyngioma in the suprasellar area [17]. At present, the pathogenesis of primary ectopic craniopharyngioma is not clear; one possible explanation for the tumor site in the present case is migration of squamous epithelial cell remnants of the obliterated craniopharyngeal canal [18].

CT and MRI are the first-choice diagnostic tools for the diagnosis of craniopharyngioma [19]. In particular, MRI shows the relationship between the tumor and adjacent neurovascular anatomy. On MRI imaging of craniopharyngioma, the lesions have uneven enhancement, with solid and cystic parts. The solid portions and cyst wall were enhanced heterogeneously. The differential diagnosis includes Rathke’s cleft cyst and xanthogranuloma of the sellar region. The MRI signal intensity of Rathke’s cleft cysts varies depending on the cyst content. However, the waxy nodules in the capsule can be of low intensity on T2WI [20]. For xanthogranuloma of the sellar region, cystic lesions that usually occur in the sellar region and/or parasellar region are difficult to distinguish based on clinical and imaging features. Typical imaging studies of xanthogranuloma of the sellar region have not been reported thus far. However, xanthogranulomas show different signal intensities on T2WI, and most of them have high signal strength on T1WI. Hemosiderin rims are found in some cases [21]. The postoperative pathological diagnosis of this case was CP with a well-differentiated squamous structure, surrounded by palisade columnar epithelium. The tumor *was* characterized by partial keratosis, and the cystic contents were accompanied by significant cystic changes.

Primary ectopic parasellar craniopharyngioma is extremely rare. One possible explanation is the migration of the remaining craniopharyngeal squamous cells. The diagnosis of this tumor is relatively difficult, leading to frequent misdiagnosis. The diagnostic process should include nasal endoscopy and imaging to better assess the tumor characteristics, and the definitive diagnosis should rely on the results of a histopathological examination. We also recommend that patients with CP undergo regular MRI follow-up after surgical resection to ensure that no relapse occurs.

## Data Availability

The data that support the findings of this study are available on request from the corresponding author, Zhiquan Jiang.

## Abbreviations

CP: craniopharyngioma
MRI: magnetic resonance imaging
CT: computed tomography
T1WI: T1 weighted image
T2WI: T2 weighted image
CTA: computed tomography angiography
